# Retraction Note: Dickkopf-1 contributes to hepatocellular carcinoma tumorigenesis by activating the Wnt/β-catenin signaling pathway

**DOI:** 10.1038/s41392-023-01475-8

**Published:** 2023-05-05

**Authors:** Rui Zhang, Hao-Ming Lin, Ruth Broering, Xiang-de Shi, Xian-huan Yu, Lei-bo Xu, Wen-rui Wu, Chao Liu

**Affiliations:** 1 Guangdong Provincial Key Laboratory of Malignant Tumor Epigenetics and Gene Regulation and Department of Hepato-Pancreato-Biliary Surgery, Sun Yat-sen Memorial Hospital, Sun Yat-sen University, 510120 Guangzhou, China; 2 Faculty of Medicine, Department of Gastroenterology and Hepatology, University Duisburg-Essen, 45147 Essen, Germany

Retraction to: *Signal Transduction and Targeted Therapy* 10.1038/s41392-019-0082-5, published online 06 December 2019

The authors have retracted this article. After publication, concerns were raised regarding suspected overlap in the cell images in Figs. 2 and 3. Specifically:

- Figs. 2f and 3f (HUH-7 Scramble) appear to contain an overlapping area.
- HepG2 Scramble in Fig. 2d and HUH-7 LV-DKK1 in Fig. 3d appear to be mirror images.
- A part of the HUH7 Scramble image in Fig. 2d appears to be enlarged and presented as HepG2 Scramble.
- The authors therefore no longer have confidence in the presented data.

All authors agree to this retraction.

